# *Notes from the Field:* Genomic and Wastewater Surveillance Data to Guide a Hepatitis A Outbreak Response — Los Angeles County, March 2024–June 2024

**DOI:** 10.15585/mmwr.mm7405a3

**Published:** 2025-02-20

**Authors:** Jordan B. Braunfeld, Bonnie L. Dao, Justin Buendia, Raiza Amiling, Cierra LeBlanc, Mirna P. Jewell, Hannah Henry, Giorgio Cosentino, Prabhu Gounder

**Affiliations:** ^1^Epidemic Intelligence Service, CDC; ^2^Los Angeles County Department of Public Health, Los Angeles, California; ^3^Council of State and Territorial Epidemiologists, Atlanta, Georgia; ^4^California Department of Public Health.

**Summary**

**What is already known about this topic?**

Routine hepatitis A virus (HAV) infection surveillance cannot detect patients who don’t seek clinical care, receive testing, or whose cases are not reported.

**What is added by this report?**

During March 12–April 18, 2024, three HAV cases were identified among persons experiencing homelessness who had a matching HAV subgenotype, indicating a potential common transmission chain. A concurrent approximately seven-times increase in HAV wastewater concentrations above baseline, when a similar number of cases were reported, demonstrated the potential for wastewater testing to detect unreported HAV transmission.

**What are the implications for public health practice?**

Genomic analysis and wastewater testing can complement traditional case-based surveillance to identify and better characterize HAV outbreaks.

In April 2024, the Los Angeles County (LAC) Department of Public Health (DPH) identified three cases of hepatitis A virus (HAV) infection among persons experiencing homelessness (PEH) through routine surveillance,* compared with one case identified among PEH during the preceding 6 months. HAV is a highly infectious fecal-oral transmitted virus that infects the liver. Most infected persons recover without sequelae, but it can rarely cause liver failure and death. Inadequate access to hygiene and sanitation services is a risk factor for HAV infection. Traditional case-based surveillance for HAV is challenging because persons with HAV are infectious for ≤2 weeks before symptom onset, and approximately 30% of infected persons do not experience symptoms. Further, health care providers might not suspect HAV infection because the early symptoms of HAV infection can be nonspecific (e.g., fever, nausea, abdominal pain, and diarrhea), and 30% of symptomatic cases will not develop jaundice to indicate hepatitis. Genomic and wastewater surveillance data helped confirm an outbreak and guide response activities by illustrating the magnitude of HAV transmission potentially undetected by case-based surveillance.

## Investigation and Outcomes

LAC DPH conducts routine hepatitis A surveillance among approximately 10 million residents in 86 of 88 cities in the county.^†^ Clinical laboratories are mandated to report positive HAV immunoglublin (Ig) M antibody (IgM anti-HAV) test results to DPH. All IgM anti-HAV–positive reports are investigated by medical records review and patient interviews to ascertain patient risk factors for HAV infection, identify close contacts for postexposure prophylaxis, and obtain specimens for molecular confirmation and phylogenetic analysis by the California DPH Viral and Rickettsial Disease Laboratory (*1*,*2*).

Wastewater surveillance for HAV in LAC began in September 2023 at two wastewater treatment plants^§^ (*3*,*4*). Plant A provides services for approximately 4 million residents, including the City of Los Angeles, and plant B for approximately 3.5 million residents.

During March 12–April 18, 2024, three HAV infections were detected among PEH through routine surveillance of patients presenting with compatible clinical signs and symptoms and laboratory findings (compared with one case among PEH during the preceding 6 months). These three cases had a matching and previously unreported HAV subgenotype IA strain^¶^ (*5*), indicating a potential common chain of transmission. A concurrent rise in HAV wastewater concentrations at plant A (Figure), which services the area where the patients lived, prompted concern for additional HAV cases not detected by surveillance and supported LAC DPH’s decision to develop an outbreak case definition** and implement enhanced case-finding measures.^††^

**FIGURE:**
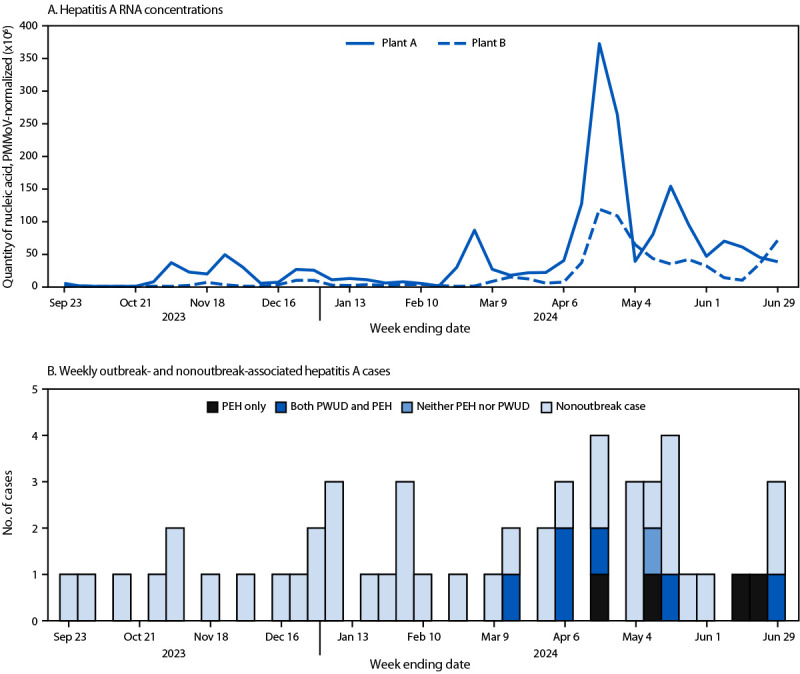
Hepatitis A RNA concentrations* measured at two wastewater treatment plants (A) and weekly outbreak-associated (N = 11)^† ^and nonoutbreak-associated hepatitis A case counts (N = 40)^§^ (B) — Los Angeles County, California, September 2023–June 2024 **Abbreviations: **ALT = alanine aminotransferase; HAV = hepatitis A virus; IgM = immunoglobulin M; PEH = person experiencing homelessness; PMMoV = pepper mild mottle virus; PWUD = person who uses illicit drugs. * Wastewater RNA concentrations are calculated as a 10-day trimmed average of RNA measurements normalized to PMMoV concentrations. ^†^ Outbreak inclusion criteria: positive anti-HAV IgM test result detected after March 1, 2024, in a person with evidence of hepatitis (ALT>200), who lived in Los Angeles County during the incubation or infectious period, and meets at least one of the following additional criteria: 1) experiencing homelessness or using illicit drugs ≤12 months before illness onset, 2) reports close contact with a PEH or PWUD during incubation period, or 3) has received positive test results for the outbreak-associated HAV subgenotype IA strain. ^§^ Date of case determined as the earliest date among the following: 1) symptom or jaundice onset date, 2) specimen collection date, 3) reported date, and 4) date elevated enzymes reported.

During April 26–June 25, eight additional outbreak-associated cases were identified (Figure). Among the 11 patients, specimens were available for sequencing from 10, and the HAV nucleotide regions sequenced were genetically indistinguishable. Among the 11 patients, six reported experiencing homelessness and illicit drug use, four reported experiencing homelessness only, and one reported neither experiencing homelessness nor illicit drug use. Nine of the 11 patients had symptoms consistent with hepatitis, and six were hospitalized. Among nine patients whose location during their infectious period was known, seven lived in the City of Los Angeles.

The maximum HAV wastewater concentrations in plants A and B in April, when seven cases (including nonoutbreak-associated cases) were reported, were three and seven times higher, respectively, than maximum concentrations recorded during September 2023–March 2024 (the baseline period, when 2–5 cases per month were reported among all LAC residents). Subsequent maximum HAV wastewater concentrations during May and June were lower than those during April. This activity was reviewed by CDC, deemed not research, and was conducted consistent with applicable federal law and CDC policy.^§§^

## Preliminary Conclusions and Actions

Compared with rises in wastewater concentration observed when HAV cases were detected during the baseline period, the rise in wastewater concentration from two wastewater plants in April 2023 was disproportionately high. This increase provided evidence for unreported transmission among a population that faces substantial structural and social barriers to accessing care.

Wastewater surveillance can be useful for detecting increases in HAV activity. However, declines in wastewater concentrations must be interpreted with caution because wastewater testing can only detect persons who become infectious and shed virus within a plant service area that is under surveillance. Persons who are exposed to HAV but become infectious in a different wastewater plant service area that does not test for HAV will not be detected by wastewater surveillance. Therefore, wastewater surveillance data must be interpreted in combination with case-based surveillance data.

Genomic and wastewater testing data supported DPH’s decision to initiate a broad community response to mitigate the outbreak. The response included mobile outreach events to offer hepatitis A vaccination to persons at higher risk for infection, county-wide public health alerts and other communications to raise awareness among clinical and homeless service providers, and increased environmental health inspections of shelters in the outbreak area. Although outbreak-associated hepatitis A cases continue to occur, recent decreases in wastewater HAV concentrations indicate that transmission might be declining.

## Footnotes

*
https://www.cdc.gov/nndss/about/index.html

†
http://publichealth.lacounty.gov/acd/procs/b73/DiseaseChapters/B73HepA.pdf

§ Wastewater testing is performed by Verily, which measures HAV RNA concentrations by using droplet digital polymerase chain reaction. Samples are taken an average of three times weekly from each plant.

¶ HAVNET strain CADPH24000422, determined through sequencing of 460 nucleotide region of the VP1/2A junction, according to HAVNET typing protocol.

** Outbreak inclusion criteria: positive anti-HAV IgM test result detected after March 1, 2024, in a person with evidence of hepatitis (alanine aminotransferase >200), who lived in LAC during the incubation or infectious period, and meets at least one of the following additional criteria: 1) experiencing homelessness or using illicit drugs ≤12 months before illness onset, 2) reports close contact with a PEH or person who uses illicit drugs during incubation period, or 3) has received positive test results for the outbreak-associated HAV subgenotype IA strain.

†† Enhanced case-finding efforts were enacted to identify hospitalized PEH before discharge to congregate settings or the streets where they might transmit the virus to others or be difficult to locate for interview or additional specimen collection. This included notices and presentations to health care providers across the county to immediately report suspected HAV infections in PEH, daily review of electronic laboratory-reported IgM anti-HAV tests, and a query of National Syndromic Surveillance Program data for mention of PEH for cross-reference to laboratory reports.

§§ 45 C.F.R. part 46.102(l)(2), 21 C.F.R. part 56; 42 U.S.C. Sect. 241(d); 5 U.S.C. Sect. 552a; 44 U.S.C. Sect. 3501 et seq.
